# “Can It Read My Mind?” – What Do the Public and Experts Think of the Current (Mis)Uses of Neuroimaging?

**DOI:** 10.1371/journal.pone.0025829

**Published:** 2011-10-04

**Authors:** Joanna M. Wardlaw, Garret O'Connell, Kirsten Shuler, Janet DeWilde, Jane Haley, Oliver Escobar, Shaun Murray, Robert Rae, Donald Jarvie, Peter Sandercock, Burkhard Schafer

**Affiliations:** 1 Division of Clinical Neurosciences, University of Edinburgh, Edinburgh, United Kingdom; 2 Scottish Imaging Network A Platform for Scientific Excellence (SINAPSE) Collaboration, Edinburgh, United Kingdom; 3 Edinburgh Neuroscience, University of Edinburgh, Edinburgh, United Kingdom; 4 Joseph Bell Centre for Forensic Statistics and Legal Reasoning, University of Edinburgh, Edinburgh, United Kingdom; 5 The Shepherd and Wedderburn Centre for Research in Intellectual Property and Technology (SCRIPT), University of Edinburgh, Edinburgh, United Kingdom; 6 Futures Forum, Scottish Parliament, Edinburgh, United Kingdom; 7 School of Social and Political Science, University of Edinburgh, Edinburgh, United Kingdom; Universidade de Brasília, Brazil

## Abstract

Emerging applications of neuroimaging outside medicine and science have received intense public exposure through the media. Media misrepresentations can create a gulf between public and scientific understanding of the capabilities of neuroimaging and raise false expectations. To determine the extent of this effect and determine public opinions on acceptable uses and the need for regulation, we designed an electronic survey to obtain anonymous opinions from as wide a range of members of the public and neuroimaging experts as possible. The surveys ran from 1^st^ June to 30 September 2010, asked 10 and 21 questions, respectively, about uses of neuroimaging outside traditional medical diagnosis, data storage, science communication and potential methods of regulation. We analysed the responses using descriptive statistics; 660 individuals responded to the public and 303 individuals responded to the expert survey. We found evidence of public skepticism about the use of neuroimaging for applications such as lie detection or to determine consumer preferences and considerable disquiet about use by employers or government and about how their data would be stored and used. While also somewhat skeptical about new applications of neuroimaging, experts grossly underestimated how often neuroimaging had been used as evidence in court. Although both the public and the experts rated highly the importance of a better informed public in limiting the inappropriate uses to which neuroimaging might be put, opinions differed on the need for, and mechanism of, actual regulation. Neuroscientists recognized the risks of inaccurate reporting of neuroimaging capabilities in the media but showed little motivation to engage with the public. The present study also emphasizes the need for better frameworks for scientific engagement with media and public education.

## Introduction

We are exposed, almost weekly, to reports of new, thrilling and increasingly fantastical applications of neuroimaging to unravel the complexities of our minds – *Am I politically right or left wing? Which of sex or money interests me most? Should I take an fMRI lie detector test? Can other people see my dreams? Can neuroimaging help me chose the right career? Can imaging identify future criminals amongst young children?* The potential for new technologies to improve the health, living and economic prospects of society quite rightly attract public curiosity [1] and subsequent media interest plays on our apparently limitless appetite for defining self and mental life [2]. However, these applications of neuroimaging have, in general, not yet been scientifically validated, or may even have gone unnoticed by experts.

Misinterpretations of neuroimaging research by the public may arise in several ways: the distortion of findings by scientists or the media to enflame interest, through commercial interests, or poor engagement of the public by researchers. Promotion of the level of public understanding of neuroscience concepts [3] suggests that distorted reporting of neuroimaging capabilities is likely to go unchallenged by the public, potentially leading to distrust through raising unrealistic expectations or unfounded ethical concerns. Public distrust of this kind has serious implications for research. Getting science a bad name may result in fewer funding opportunities and restrictive regulations on neuroimaging used in basic research or for medical care and thus also harm the public. It is reasonable to suggest that scientists have a responsibility to ensure the effective communication of their discoveries so that the public can critically assess the potential dangers and benefits inherent in new technologies, although lack of opportunity and training for engaging the media and public is still perceived to be a barrier to more effective public communication amongst neuroimaging professionals [4].

Motivated by the need for evidence-based solutions to these challenges, we surveyed the public for their thoughts on the current uses, communication of, and need for regulation of, neuroimaging research. In parallel, we also surveyed experts in neuroimaging to provide a benchmark of the scientifically accepted uses and identify where public and professional opinions might differ.

## Methods

### Survey Design

We designed two short questionnaires, one for the public and one for neuroimaging experts, suitable for delivery online or on paper. The public and expert surveys were modified to reflect the respondents' likely knowledge of and degree of personal relevance in determining their opinion on each issue. There were 10 questions in the public survey and 21 questions in the expert survey (abbreviated in Table 1), with a mixture of multiple-choice and forced-choice formats. We included a short introduction to provide an overview of the newer applications of neuroimaging. We themed pages into inquiries about personal information (“About You”), awareness of neuroimaging methods (“About Neuroimaging”), and scientific communication (“About Communication”). Respondents were informed that their responses would be anonymous and would remain confidential. Respondents to the Public Survey were also asked for their age, general occupation (student, manual, skilled, administrative, professional, etc) and highest level of educational attainment. Respondents to the Expert Survey were also asked for their country of residence, profession, capacity in which they used neuroimaging and years of usage.

**Table 1:** List of abridged public and expert survey questions.

Abridged Survey
**Public**
How familiar are you with brain scanning methods?
To what extent do you think neuroimaging can achieve the following?
Would you be comfortable having your brain scan used for the following? (e.g. employment screening/marketing research)
If brain imaging is used for the purposes above, how concerned are you about the following? (e.g. data storage/privacy)
Can you remember having seen or heard information about Brain Imaging in the following places? (e.g. online/newspaper)
If you feel that brain imaging should be regulated to protect the public from its potential misuse, then how do you feel that this should be done? (e.g. law/self-regulation)
**Expert**
Please indicate which methods of neuroimaging outside of tradition uses are you aware of?
Where would you choose to seek information about uses of neuroimaging outside traditional uses?
Do you think neuroimaging can presently achieve the following? (e.g. diagnose mental illness/lie-detection)
What do you think may be the future effects of the widespread use of neuroimaging? (e.g. innovative applications/change legal system)
Rate in ascending order what you think would be the best strategies to encourage use of neuroimaging within the limitations of its capabilities. (e.g. law/public education)
How important do you think is it that neuroscientists and clinical researchers communicate with the public about their research?
How effective do you think the following incentives would be to encourage researchers to engage with the public? (e.g. funding requirement/public exposure)
Do you think neuroimaging research findings are, in general, accurately portrayed in the media? Why? (e.g. poor journalism/poor media skills by scientist)
What do you think may be the future effects of the widespread use of neuroimaging? (e.g. new funding opportunities/over-regulation)

The survey was posted online, using the Survey Monkey Pro web survey template, between 31^st^ May and 15^th^ December 2010. Links to the Public Survey were targeted at the UK general public to match a recent systematic review of media coverage of neuroimaging which focused on the UK media [5]. The Expert survey was not restricted to the UK but we only provided an English version. The Public Survey was promoted through being mentioned in national newspapers (e.g. *The Guardian*, *The Glasgow Herald*), websites (e.g. *BBC News*, *The Times Online*), tweets (e.g. *The Times Science*, *The Guardian Science*), and science blogs (e.g. *Law and Neuroscience*, *Neurophilosophy*) as well as to attendees at meetings (e.g. Scottish Parliament Futures Forum) and societies for public engagement (Beltane http://www.publicengagement.ac.uk/about/beacons/edinburgh-beltane). The Expert Survey was distributed via university mailing lists (e.g. Edinburgh Neuroscience, Institute of Cognitive Neuroscience, SINAPSE), professional interest websites (e.g. SINAPSE, Oxford Neuroscience blog, British Society of Neuroradiologists, International Society of Magnetic Resonance in Medicine, British Institute of Radiology, British Neuroscience Association) and individual imaging research centre websites) to target individuals who routinely used neuroimaging methods (*for further information, see Table S1*). Ethics approval was not sought. A level one self-assessment indicated that there was no risk to the respondent or the researcher.

### Statistical Analysis

We used descriptive statistics to characterize the composition of the sample, t-tests to examine differences between the samples and chi-squared tests to analyse main effects (*p*<0.05). All available data were included. Percentages are based on samples of completed response, as opposed to the entire potential sample. After main effects were examined, we examined for trends within demographic profiles. The public sample was divided into groups based on scientific interest. The expert sample was divided based on the capacity in which they used neuroimaging technology (e.g. medical, academic, marketing research, etc).

## Results

### Characteristics of Sample

There were 963 respondents, of whom 660 completed the Public Survey and 303 the Expert Survey. Most respondents to the Public Survey were aged between 26 and 40 (42%) and the majority described themselves as professionals (60%), students (16.5%), administrator (9.8%), not working (9.8%), skilled manual (2.7%) or manual workers (1.1%). The highest educational attainment was university undergraduate degree (30%), a Masters degree (21.3%), a PhD (15.9%), a professional qualification (15.5%), minimum school leaver's exam (8.9%) or final school leaver's exam (8.1%). Most respondents to the Expert Survey were neuroscientists (27%), followed by psychologists (17%), neuroradiologists (12.5%), psychiatrists (12.5%) or medical physicists (10.5%); had been using neuroimaging for 5–10 years (31%), <5 years (28%) or 10–20 years (26%); and were based in Scotland (34%), elsewhere in the United Kingdom (32%), Europe (16%) or the United States (15%).

### Public Survey Response Profile

Almost half the sample reported themselves as at least ‘a little’ aware of neuroimaging uses (47%), followed by ‘quite aware’ (26%), ‘no awareness’ (17%) and ‘very aware’ (10%). A subgroup (*n* = 39, 5%) who said that their professions involve neuroimaging and may therefore not be representative of the public profile was identified, but no differences were found between the groups (*p*>0.05).

**Figure 1:**
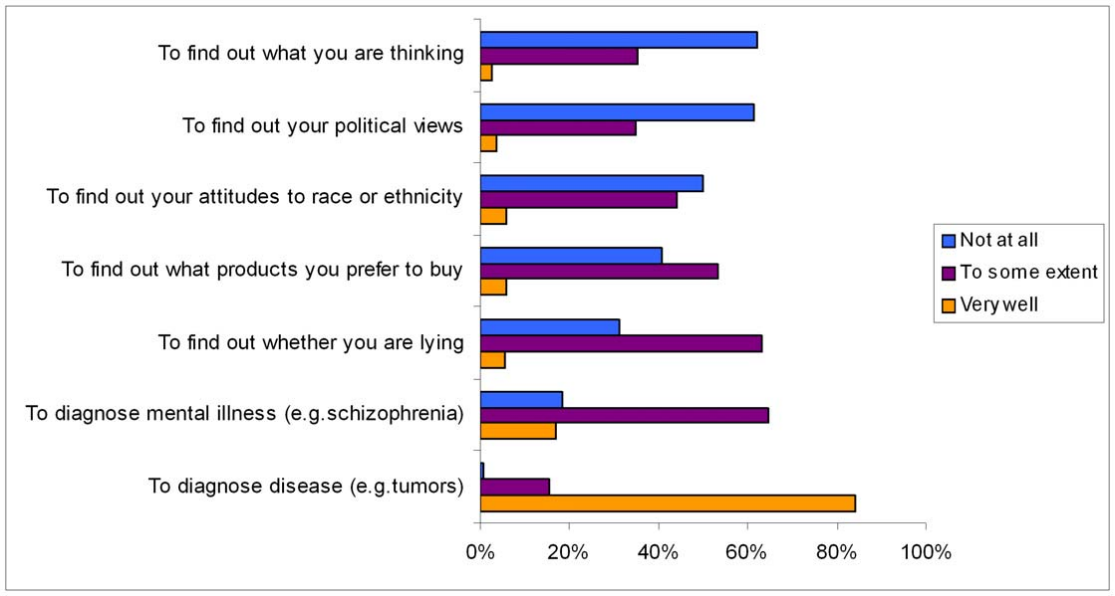
Responses from members of the public to how well neuroimaging can achieve various aims.

Respondents thought that neuroimaging could diagnose brain diseases such as tumors (‘very well’ 84%, ‘to some extent’ 15%), but few had the same confidence in use of neuroimaging to diagnose mental illness (‘very well’ 17%, ‘to some extent’ 64%), some thought that neuroimaging could detect lies (‘very well’ 5.6%, ‘to some extent’ 62%) or consumer preferences (‘very well’ 6%, ‘to some extent’ 53%), but had less confidence in detecting racial attitudes (‘to some extent’ 43%, ‘not at all’ 49%), political views (‘to some extent’ 34%, not at all 61%) or reading minds (‘to some extent’ 34%, ‘not at all’ 61%), Figure 1.

**Figure 2:**
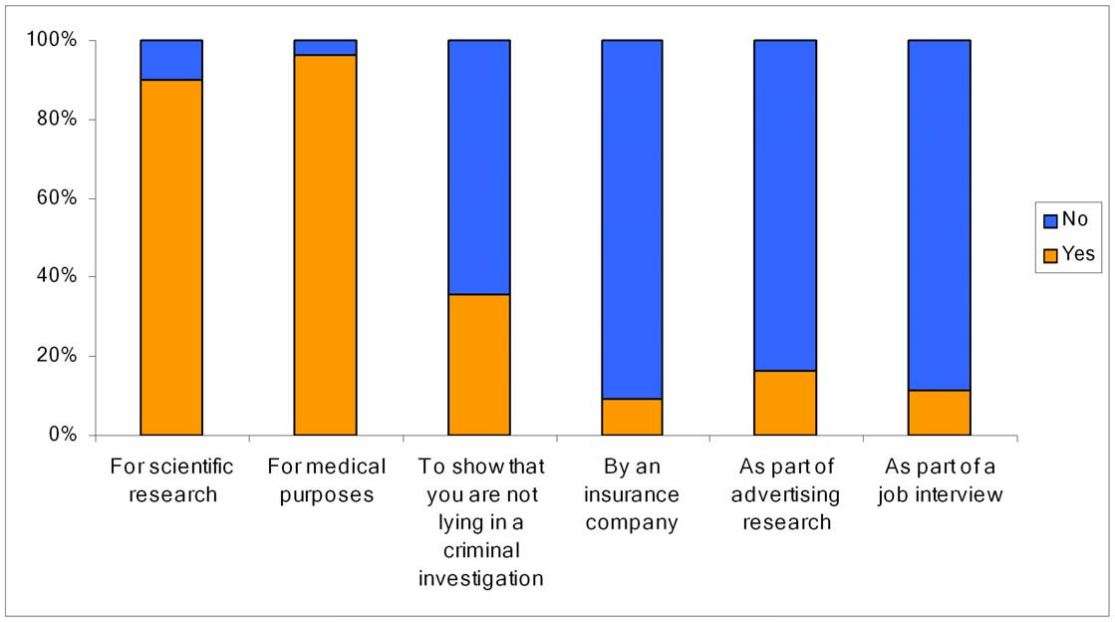
Responses from the public on how comfortable they would be to have their brain scanned for various purposes.

Most respondents reported that they would be comfortable having their brain scanned for medical purposes (96%), for scientific research (90%), but were less inclined as part of a criminal investigation (36%), for insurance purposes (9%), for marketing research (16%) or as part of a job interview (11%) Figure 2.

**Figure 3:**
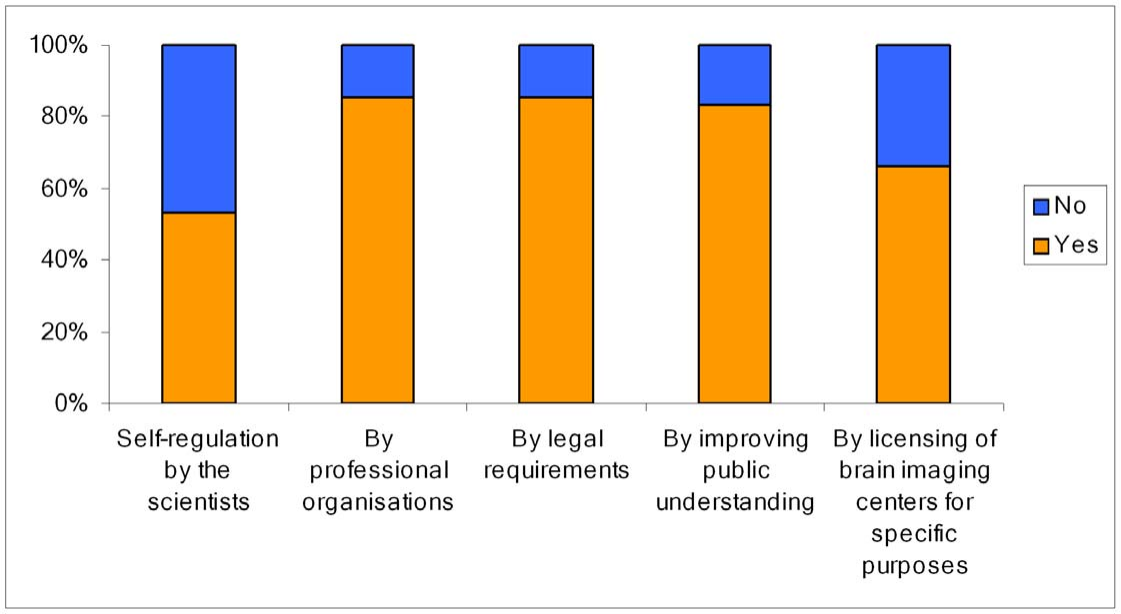
Responses from the public on preferred strategies for managing uses of brain imaging.

Most respondents were ‘very’ or ‘quite’ concerned about the confidentiality and storage of scans (82%) and that ‘people would know what they were thinking’ (55%), being forced to have a scan (70%) or that there would be ‘something wrong with their brain’ (57%). Most were ‘not at all ‘or’ only a little ‘worried that having a scan would make them vulnerable to thought control (61%), Figure 3.

Most respondents reported that newspapers (65%) and popular science publications (64%) were the most common sources for information about neuroimaging, followed by television documentary (62%), television or film fiction (59%) and online sources (46%). Of those who reported using online sources, twitter and blogs were the most popular. When asked how often they encountered information on brain imaging, responses were highly varied with about a third reporting a little (i.e. once/twice in the last year; 35%), often (i.e. one/twice in last 6 months; 29%) and very often (i.e. once/twice a month; 30%).

When asked about the regulation of brain imaging, most public respondents chose legislation (85%) and professional regulatory bodies (86%), followed by increased public awareness (84%). The least favoured response was the self-regulation of brain imaging research by the scientists themselves (53%), followed by the licensing of the scientists to conduct neuroimaging research (66%).

### Expert Survey Response Profile

Most expert survey respondents reported using neuroimaging for neuroscience research (66%), followed by clinical research (47%), medical diagnosis (35%), neuromarketing (4%), forensic purposes (3%) and security purposes (1%). The majority of respondents were aware of the use of neuroimaging outside traditional research (86%), particularly for neuromarketing (67%) and lie-detection (68%). Moderate awareness was reported for legal uses (48%), neuroaesthetics (56%), cognitive enhancement (55%) and mind reading (45%). Few were aware that neuroimaging was being used for security purposes (11%).

Most respondents report specialist peer-reviewed literature as their main source of information on neuroimaging (84%), followed by general literature (65%), popular science media (50%), research conferences (54%), science blogs (25%), newspapers (25%) and popular science websites (23%).

**Figure 4:**
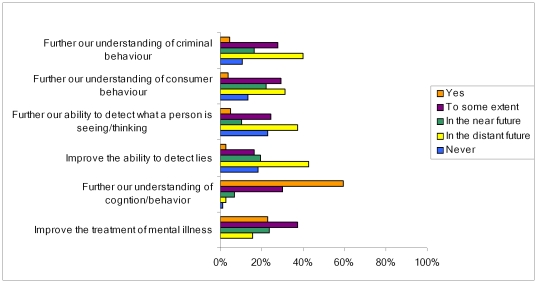
Expert opinions on capabilities of neuroimaging.

Most respondents thought that neuroimaging could improve the understanding of cognition (‘yes’ 59%, ‘to some extent’ 30%), or improve treatment of psychiatric illness (‘yes’ 23%, ‘to some extent’ 37%, in the ‘near or distant future’ 39%). However, most experts thought that the ability of neuroimaging to detect lies, read minds, understand consumer or criminal behaviour was largely a thing of the future or unlikely ever to occur (Figure 4): 29% of respondents believed neuroimaging currently had mind reading potential ‘to some extent’, but 38% thought this would only be possible ‘in the distant future‘ and 23% ‘never’; 33% thought that neuroimaging could contribute to marketing research at present at least ‘to some extent’, but 45% thought this would only be possible in the ‘distant future’ or ‘never’; 40% thought that neuroimaging might improve understanding of criminal behaviour in the ‘distant future’.

When asked how often neuroimaging evidence has been presented in U.S. courts in the last 5 years, most believed it was between 10 and 30 times (24%), followed closely by 6–10 times (22%) and between 1 and 5 times (23%). The lowest rates were reported for greater than 100 times (15%), between 30–60 times (8%) and between 60 and 100 times (8%).

**Figure 5:**
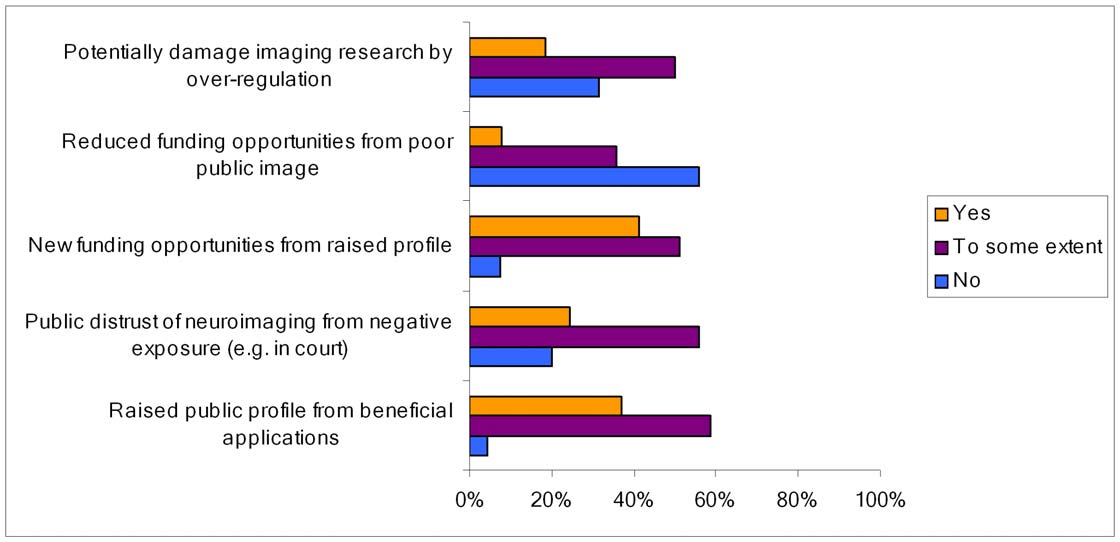
Responses from experts on the expected effects of widespread use of neuroimaging.

Most respondents thought that wider uses of neuroimaging would, to some extent, lead to *development of new applications*, *changing the legal system*, *changing views on responsibility for criminal behavior* and *enhancement of mental abilities*, although *changing the legal system* and *enhancing mental abilities* were considered the least likely amongst these options (Figure 5). These views were offset by most respondents considering that any of these possibilities were of low likelihood: *innovative applications* (45%), *cognitive enhancement* (46%), *change views of criminal responsibility* (34%) and *change the legal system* (47%).

In terms of strategies for regulating use of neuroimaging, respondents ranked professional guidelines first (66% ranked first or second choice), followed by more funding for interdisciplinary projects integrating neuroscience and law (50%) and improved public awareness (51%), with legal regulation (16%) and self regulation (15%) being placed last.

A substantial majority (87%) felt that neuroimaging was not accurately portrayed in the media. When asked why, most cited poor journalism (54%) as either a quite or very important factor, followed by exaggerated results by researchers (51%), a lack of effective scientific communication (38%) and poorly conducted research (44%). Most respondents believe that increased exposure to neuroimaging research would raise its public profile as a beneficial technology (96%), whereas 80% foresaw a public backlash on the widespread use of the technology. 92% predicted an increase in funding opportunities, but 44% anticipated the opposite trend. 69% feared the over regulation of neuroimaging in research.

Having said that, although most respondents thought it was important to communicate with the public (69%), most do not regularly engage with the media (52%). Of those that do communicate their results to the public, most used media and peer-reviewed publication to do this (78%), followed by public seminars (49%), the internet (39%) and television (28%). Among those that did not engage with the public, the majority gave little opportunity (58%), little incentive (38%) and distrust of media (27%) as their main reasons, followed by a lack of public speaking training (15%) and a fear of criticism (5%). When asked how this situation might be improved, most rated as ‘quite’ or ‘very effective’: professional credit for public engagement (75%), making public communication a funding requirement (72%), increased public exposure of research (76%), and outlets for a career in the media (25%).

## Discussion

This survey of members of the public and neuroimaging experts revealed a range of similarities and differences of opinions. About a third of the public saw articles on neuroimaging in the media once or twice a month indicating, in line with previous research [6], a surprisingly high penetrance of articles on the topic in the ‘public eye’. Recent widely publicised uses of neuroimaging include predicting future criminal behaviour in young children [7], determining future career choices [8], lie detection [9], identifying terrorists [10] and detection of guilt [11], [12], to name but a few [5], [13], [14]. While it may be too early to say if these claims will ever hold substance or be applicable reliably in practice, there is currently an inverse association between the quality of study reporting in the media and the remoteness of the purpose to which the imaging was being put with respect to established medical or research uses. News reports on commercial uses were particularly unlikely to include information relevant to study quality. [13].

Although the public and experts were generally in agreement about some points, e.g. both had little faith in uses of neuroimaging in non-medical applications and both agreed on some methods to use for regulation, the results also suggest that extensive media coverage has moderated viewpoints and awareness differentially among the public and experts. The public were concerned about data protection issues. The experts had little awareness of how often neuroimaging had been used in court in the USA (the actual number of times is well in excess of 100 times in the last few years) and one in three experts reporting no familiarity with neuromarketing or commercial lie-detection, although the public were aware of these uses. Although the experts thought that neuroimaging results were not well communicated in the media, and recognised the dangers of public loss of trust, few experts seemed motivated to improve the situation.

Who can say which, if any, of the current legal, commercial or governmental uses of neuroimaging may become in future years routine, reliable tools? Neuroimaging is a rapidly evolving discipline; insights gained from well-conducted neuropsychological imaging research are in turn influencing knowledge of human behavioural traits on which many of society's attitudes are based. Consequently, it is entirely possible that society will need to adjust its views on behaviour, culpability, consciousness, etc. in future. However, a major focus of the present study was to determine how the public perceived the current capability of neuroimaging. Limited confidence in diagnosing psychiatric illness, lie-detection and neuromarketing was found, and to a far lesser degree in revealing racial and political attitudes and inner thoughts. Interestingly, the evidence base that complex social behaviours, such as racial attitudes and basic forms of mental representation, can be inferred using neuroimaging (for a review see [15]), is arguably superior to existing support for neuromarketing tools [16]. This may suggest a natural scepticism amongst the public about the capabilities of different applications of neuroimaging.

Interestingly, experts appeared more optimistic about the potential of neuroimaging applications compared to the public. On the one hand, public scepticism about neuroimaging could protect them against media misrepresentations. On the other hand, inclusion of complex brain images in media reports introduces an element of “photorealism”[15], [17] and makes the information being presented much more persuasive to the public than the same information presented without images [18]. Confidence among experts means that there is no shortage of neuroscientists to provide affirmation of neuroimaging capabilities that can then unfortunately be taken out of their experimental context and used to support other applications of neuroimaging. Media engagement by neuroscientists needs to be done responsibly and placed in the context of scientific evidence in a way that is easily understood.

One of the clearest trends identified in the survey is the public disapproval of the use of neuroimaging in non-medical or scientific settings, such as in marketing research, and in employment screening. Indeed, respondents who made specific comments cited moral and ethical grounds as explanations for this standpoint (e.g. *“Private sector does not have the right to that information”*) especially with regards privacy and human rights (e.g. *“Anything that is voluntary is acceptable. Mandatory brain scans are not…”*). The public distrust of other technologies used to extract sensitive information for non-health related uses has been echoed in a public survey on the acceptability of genetic testing in insurance and employment settings [19].

The results indicate that experts are less alarmed about the ethical implications of wider uses of neuroimaging than are the public. Instead, concerns were expressed about a possible public backlash against exploitive uses of neuroimaging with the risk of subsequent overregulation. Experts therefore appear receptive to public concerns as they relate to progress of the field. However, most experts do not engage regularly with the media. In line with previous findings [4], [20], our results indicate that neuroscientists lack opportunity or willingness to respond to the media. The experts favoured establishment of professional guidelines, followed by increased public awareness and interdisciplinary projects, with self-regulation and legal regulation being least popular. In contrast, the public preferred professional guidelines, legal regulation and improved public awareness, with self regulation by scientists receiving least support. Combined with the public's concerns about data protection issues, these subtle differences of emphasis (e.g. on ranking of legal regulation) suggest a lack of public confidence in scientists to effectively and ethically conduct research and curb misuse, opting instead for external control. Indeed other research indicates a low professional opinion of existing research ethics committees' ability to tackle emerging ethical challenges [21], which , taken with the experts' own low opinion of the accuracy of media reporting of neuroimaging identified in our survey, suggests that the science is in serious danger of falling into disrepute.

Both the public and expert groups showed support for public education as an integral component of future strategies to improve scientific communication. Responses strongly suggest that the hesitancy of experts to engage with the media may arise due to fear of misrepresentation. When asked what the main causes of media distortion were, the majority of experts cite poor journalism. This is in contrast to a recent systematic review of media articles on neuroimaging where irresponsible scientific engagement was frequently reported [5]. Each sector seems to be blaming the other for exaggerated claims and poor communication.

These findings converge on the view that the media moderates differences between the public and experts in the way they absorb information, perceive potential dangers and rate solutions to communication challenges and ethical concerns associated with advances in neuroimaging. Nascent emerging applications of neuroimaging carry profound ethical considerations for society [14], a trend that demands increasing focus from empirical research for the development of effective strategies. Further work is needed to examine issues surrounding the implementation of specific strategies before translating them into large-scale international approaches. As technologies advance and the media become more attentive to these discoveries, such frameworks could provide a template for other fields in science to tackle new challenges in the dissemination of research to the public.

## Supporting Information

Table S1 Record of Survey Destinations.
(DOC)
