# Supplementary material for: “Can It Read My Mind?” – What Do the Public and Experts Think of the Current (Mis)Uses of Neuroimaging?
Source: PLoS One. 2011 Oct 4;6(10):e25829. doi: 10.1371/journal.pone.0025829 (PMC3186771; doi:10.1371/journal.pone.0025829)
Supplement: Table S1 — Record of Survey Destinations. (DOC) [file pone.0025829.s001.doc]

Table S1. Record of Survey Destinations

(some destinations reached both expert and lay audiences and contained information on how to access both surveys )

ONLINE

Lay Survey

*Article Ant*

*BBC news* website

*British Neuroscience Association* website

*Centre for Cognitive Neuroimaging,* Edinburgh website

*Edinburgh Beltane,* Public Engagement website

*Edinburgh Neuroscience* website

*Epiphenomenon* blog

*EUSci* website

*Facebook* - Jane Wyllie, Garret O’Donnell, Janet de Wilde personal pages

*Facebook* - Edinburgh Neuroscience page (310 fans)

*Guardian-science* twitter feed

*Health and Science* blog

*Humanists4Science*

*Institute for Advanced Studies*, Strathclyde website

*MacArthur Foundation’s Law and Neuroscience* blog

*MOCOST* twitter feed

*Nexxus* e-bulletin

*Oxford Neuroscience* blog

*Science and Religion today* blog

*Scotland’s Future Forums*, Scottish Parliament website

*See Me Scotland* website (Mental health support group)

*Sense about Science* website

*SFC Brain Imaging Research Centre* website

*Times science* twitter feed

*TimesOnline science* blog

**Expert Survey**

*British Journal of Psychiatry* profiles

*British Neuroscience Association* website

*The British Society of Neuroradiologists* website

*Centre for Cogntive Neuroimaging* website

*Edinburgh Neuroscience* website

*Epiphenomenon* blog

*Humanists4Science*

*MOCOST* twitter feed

*MacArthur Foundation’s Law and Neuroscience* blog

*Neuroethics and Law* blog

*Nexxus* e-bulletin

*Oxford Neuroscience*

*SINAPSE* website

*SFC Brain Imaging Research Centre* website

WRITTEN PRESS

**Lay Survey**

*Daily Telegraph*

*British Neuroscience Association* bulletin

*SINAPSE* newsletter

Expert Survey

*British Neuroscience Association* bulletin

DISTRIBUTION LISTS

Lay Survey

*Centre for Cognitive Ageing and Cognitive Epidemiology* email list
*Edinburgh Neuroscience* email list (1000)

*Edinburgh Neuroscience Alumni* email list (50)

*Medical Consultant Staff*, Edinburgh University email list

*SINAPSE* email list (590)

*UCL Neuroscience* email lists: *ICN (institute of cognitive neuroscience)*, ION (neurology), IBN (behavioural neuroscience) and Cognitive, brain & perceptual sciences unit in psychology.

*SPM* email list

Expert Survey

*Centre for Cognitive Ageing and Cognitive Epidemiology* email list
*Edinburgh Neuroscience* email list (1000)

*Edinburgh Neuroscience Alumni* email list (50)

*Medical Consultant Staff*, Edinburgh University email list

*Neuromarketing/Lie-detection neuroscientists* sourced by Garrett O’Connell

*SINAPSE* email list (590)

*UCL Neuroscience* email lists: *ICN (institute of cognitive neuroscience)*, ION (neurology), IBN (behavioural neuroscience) and Cognitive, brain & perceptual sciences unit in psychology.

*SPM* email list
